# Evaluation of Medical Malpractice in Primary Healthcare: A 20-Year Retrospective Analysis of Supreme Court Decisions

**DOI:** 10.5152/eurasianjmed.2026.261360

**Published:** 2026-05-11

**Authors:** Gülşen Taşdemir Sancı, Abdulkadir Sancı, Talip Vural

**Affiliations:** 1 Department of Family Medicine, Kafkas University Faculty of Medicine, Kars, Türkiye; 2 Department of Forensic Medicine, Directorate of Kars Forensic Medicine Branch, Kars, Türkiye; 3 Department of Forensic Medicine, Atatürk University Faculty of Medicine, Erzurum, Türkiye

**Keywords:** Court of cassation decisions, expert witness, malpractice, nursing, primary health care

## Abstract

**Background:**

Medical malpractice occurring in primary health care settings may have significant consequences for patient safety, quality of care, and the legal liability of health care professionals. This study aimed to retrospectively examine malpractice cases alleged to have occurred in primary health care institutions in Türkiye and adjudicated by the Court of Cassation, in order to describe the types of errors, case outcomes, characteristics of the judicial process, and decision patterns.

**Methods:**

Using the official decision search system of the Presidency of the Court of Cassation of the Republic of Türkiye, 22 decisions meeting the inclusion and exclusion criteria were identified and included. Data were evaluated with respect to the type of action (civil compensation vs. criminal), the profession of the defendant, the alleged type of error, the outcome of the incident, the status of the expert report, and judicial decisions. Statistical analyses were performed using IBM SPSS Statistics for Windows, Version 22.0 (IBM SPSS Corp.; Armonk, NY, USA).

**Results:**

Of the decisions reviewed, 54.5% concerned compensation claims and 45.5% were criminal proceedings. Nurses constituted the most frequently prosecuted professional group (40.9%), and the most common malpractice allegation was incorrect injection administration (31.8%). The vast majority of incidents occurred in health centers/family health centers.

**Conclusions:**

In primary health care, medical malpractice allegations primarily cluster around nursing practices and injection procedures. Strengthening training, supervision, record-keeping systems, and intra-team allocation of responsibilities will play a critical role in reducing malpractice in primary care.

**Main Points**

- In primary health care services, lawsuits are filed most frequently against nurses, followed by family physicians and then general practitioners.
- The most common alleged act is intramuscular injection administration.
- The most frequent permanent injury identified is sciatic nerve damage.
- The incidents giving rise to litigation most often occurred in health centers/family health centers.
- Strengthening training, supervision, record-keeping systems, and intra-team allocation of responsibilities will play a critical role in reducing medical malpractice in primary care.

## Introduction

Primary health care constitutes the foundation of the health system and represents the first level of care to which individuals apply. At this level, essential services—such as prevention, diagnosis, treatment, and rehabilitation—are delivered in an integrated manner. Family health centers, community health centers, tuberculosis control dispensaries, migrant health centers, village health houses, and similar institutions are included within the scope of primary health care services. In addition, because access is easy, widespread, and can generate a high impact at relatively low cost, primary health care is indispensable for health systems.^1^ Medical malpractice, which is also referred to as professional negligence, is a major problem with serious potential consequences that directly affects both patient safety and the quality of health care, and it has ethical as well as legal dimensions. The term “malpractice,” derived from Latin, combines male (“wrong”) and praxis (“practice”). In general, it is defined as harm to a patient resulting from ignorance, inattention, inexperience, lack of due care, or unethical behavior during the provision of health services.^2^^-^^4^

In Türkiye, a marked increase has been observed in the number of lawsuits related to medical malpractice in recent years. This increase can be tracked particularly through cases brought before the Court of Cassation on appeal. One of the most notable issues in these cases is the difficulty in establishing fault, the inadequacy of expert reports, and the frequent reversal of first-instance court decisions by the Court of Cassation.^5^ Accordingly, decisions of the Court of Cassation provide important insights not only as a higher judicial review body but also into judicial practice concerning medical errors and the functioning of the expert-witness mechanism.

In primary health care, situations such as misdiagnosis, delayed referral, incomplete medical records, and medication administration errors may be encountered frequently. However, in the literature, comprehensive studies that systematically examine medical errors specific to primary health care within the framework of Court of Cassation decisions are quite limited. Yet, medical errors encountered in primary care may reflect a combination of multiple problems, including deficiencies in diagnostic and follow-up processes, incorrect or delayed referral decisions, prescribing errors, and communication problems between health care professionals and patients. Although such errors do not always result in severe outcomes such as death or permanent disability, they may still cause harm that is difficult to remedy for patients and may undermine trust in the health system.

The main aim of this study is to retrospectively examine medical malpractice cases related to primary health care practices in Türkiye that were adjudicated by the Court of Cassation, and to elucidate the characteristics of these errors, their underlying causes, and how they are assessed during judicial proceedings. In doing so, the study seeks to shed light on allegations of malpractice encountered in primary care practice and to contribute both to preventive health care approaches and to expert-witness standards in judicial processes.

## Material and Methods

In this study, cases concerning alleged medical malpractice in primary health care institutions that were brought before the judiciary and subsequently appealed to the Court of Cassation following objections to the decisions or judgments rendered after review by courts of first instance were examined. To this end, the advanced search module of the official website of the Presidency of the Court of Cassation of the Republic of Türkiye (https://karararama.yargitay.gov.tr) was used. The keywords “Family Health Center,” “Health Center,” “Community Health Center,” “Tuberculosis Dispensary,” and “Maternal And Child Health And Family Planning Center” were searched separately in combination with the term “fault” (“kusur”). Court of Cassation appellate decisions adjudicated between January 1, 2004 and December 31, 2023 were reviewed.

Decisions issued by the Court of Cassation between 2004 and 2023 were screened. Of the 284 decisions retrieved through searches combining each primary care keyword with “fault,” those unrelated to primary health care institutions, not classifiable as medical malpractice, of a different legal nature (e.g., administrative acts, labor law disputes, assault/violence cases, civil servant disciplinary investigations), not involving a medical intervention, or duplicative decisions concerning the same incident were excluded. Ultimately, 22 decisions were identified as involving alleged medical malpractice occurring in primary health care settings.

For each of the 22 cases, the following variables were recorded and examined: the medical practice at issue, the alleged basis of negligence, the outcome of the incident, presence of permanent injury, the type of health care institution, the party bringing the action and the defendant, whether an expert opinion was obtained, the decision of the court of first instance and the decision of the Court of Cassation, and the time elapsed between the incident date and the Court of Cassation decision. Statistical analyses were conducted using IBM SPSS Statistics for Windows, Version 22.0 (IBM SPSS Corp.; Armonk, NY, USA). Data were presented as frequencies and percentages. Differences between categorical variables were assessed using the chi-square test. Statistical significance was set at *P *<0.05

Informed Consent: Informed consent was waived by the Ethics committee due to the retrospective nature of the study.

Ethics Committee Approval: The current study was approved by the Ethical Committee of the Medical Faculty of Kafkas University (Approval no: 792; Date: November 24, 2025). All methods were performed in accordance with the relevant guidelines and regulations outlined in the Declaration of Helsinki.

## Results

In this study, 22 Court of Cassation decisions adjudicated between 2004 and 2023 and meeting the predefined eligibility criteria were reviewed. Of these decisions, 54.5% (n = 12) concerned compensation claims. When the interval between the date of the alleged malpractice and the date of the Court of Cassation decision was examined, the mean time to adjudication was 6.4 years (min = 1, max = 10) in criminal cases and 7.6 years (min = 2, max = 20) in compensation cases.

The applicants were most commonly physicians and relatives of patients. Nurses constituted the most frequently prosecuted professional group (40.9%). With regard to the alleged grounds for malpractice, 31.8% of the case files (n = 7) involved an incident attributable to incorrect injection administration. In 31.8% of cases (n = 7), the incident resulted in injury; in nearly all injured individuals (n = 6), permanent sciatic nerve damage was identified. Most incidents (86.4%, n = 19) occurred in health centers/family health centers. In 45.5% (n = 10) of cases, the court of first instance held that the defendant had committed a faulty medical practice, and in 59.1% (n = 13) an expert opinion was requested. The Court of Cassation reversed 68.2% (n = 15) of first-instance decisions (Table 1).

A statistically significant association was found between the ground for alleged malpractice and the decision of the court of first instance (*P* = .021); however, no statistically significant association was observed with the Court of Cassation decision (*P* = .474) (Table 2).

A statistically significant association was found between the defendant and the decision of the court of first instance (*P* = .009); however, no statistically significant association was observed with the Court of Cassation decision (*P* = .145) (Table 3).

No statistically significant differences were found with respect to whether an expert opinion was obtained in relation to either the decision of the court of first instance (*P* = .096) or the decision of the Court of Cassation (*P* = .899) (Table 4).

## Discussion

As the court of last resort, the Court of Cassation plays a critical role in malpractice litigation. In this study, medical malpractice case files that were brought before the judiciary on the allegation that the events occurred in primary health care institutions and that were ultimately adjudicated by the Court of Cassation were examined. The findings indicate that malpractice allegations arising from primary care constitute a very small proportion of all Court of Cassation decisions. This may be explained by the relatively lower-risk nature of services provided in primary care, the limited and less invasive character of interventions, and the functioning of the referral pathway as a factor that may reduce litigation risk.

A study published in 2022 reported that 83.1% of lawsuits filed between 2015 and 2020 on the grounds of alleged malpractice were compensation claims, and similar studies conducted in 2018 and 2021 likewise found that compensation cases predominated.^5^^-^^7^ Consistent with the literature, the study showed that compensation cases were more frequently brought before the Court of Cassation and that, when the time from the alleged incident to the Court of Cassation decision was compared, compensation cases took approximately 1 year longer on average than criminal cases. Thus, incorporating malpractice allegations into judicial proceedings may take a substantial amount of time, which in turn may hinder the timely and adequate recovery of compensation sought by patients or their relatives.

In a study evaluated by the General Council of Forensic Medicine between 1996 and 2001, 62.1% of health care professionals accused of malpractice were physicians; a similar study conducted in the Department of Forensic Medicine at Çukurova University reported that 92.3% of malpractice allegations targeted physicians; and another study in Kayseri found that specialist physicians were prosecuted for alleged malpractice at a rate of 83.9%.^8^^-^^10^ In the study, although physicians accounted for 31.8% of defendants, nurses were most frequently prosecuted (40.9%). In primary care, nurses often have responsibilities such as routine care, medication administration, and documentation; moreover, due to communication gaps and incomplete records, nurses may become the first point of contact in litigation.

In 1 study of cases involving malpractice allegations, 66.7% of complaints were related to errors in standard care and 22.8% to medication errors; in a study examining allegations in obstetrics and gynecology cases resulting in death, 47.5% of complaints were attributed to lack of due care; and another study focusing on nurses reported that medication administration errors ranked first, accounting for 47% of allegations.^11^^-^^13^ In the study, the most common allegation was incorrect injection administration. This difference may reflect the focus of the study on primary care institutions rather than higher-level facilities and the prominent role nurses assume in administering injections. Such errors may pose serious risks to patient health and are expected to be preventable through strengthened training and supervisory mechanisms.

Review of the Court of Cassation decisions showed that nearly all allegations related to incorrect injection administration resulted in permanent sciatic nerve injury. Injection administration is among the most frequently performed nursing procedures, and intramuscular injections are known to cause complications—such as abscess, hematoma, and neurological sequelae—depending on the characteristics of the injection site.^14^ Although neuropathies are among the most important complications following injection, the most common—sciatic neuropathy—is characterized by clinical findings such as foot drop, loss of flexion and extension of the toes, dysesthesia, chronic leg and foot pain, and sensory loss in the foot.^15^^,^^16^ Because incorrect injections may arise from inadequate training and deficiencies in safe injection techniques, ensuring that health care workers learn and apply safe injection techniques in detail may substantially reduce the risk of permanent nerve injury. In addition, a thorough understanding of the anatomy of the injection site and the use of ultrasound during the procedure in patients presenting to primary care for injection may help prevent neurological injury.

A study encompassing all health institutions in Türkiye reported that malpractice allegations were most frequently reported in private hospitals, followed by public hospitals, and that 0.4% occurred in family health centers.^17^ In the study, the alleged malpractice incidents most commonly occurred in health centers/family health centers, and less frequently in tuberculosis dispensaries. No Court of Cassation adjudications related to malpractice allegations were identified for other primary care settings such as community health centers, migrant health centers, or village health houses. This may be attributable to the high patient volume at health centers/family health centers, the diversity of medical procedures and case mix, and differences in documentation and oversight mechanisms. In particular, continuous training and quality assurance measures in family health centers and tuberculosis dispensaries may minimize malpractice risk.

In a study including 175 first-instance court decisions, 22.3% of defendants were found liable for negligent homicide, 5.1% for negligent injury, and 1.7% for abuse of office, totaling 29.1% of cases in which faulty medical practice was established.^5^ Another study reported that malpractice was accepted and judgment rendered in 22.3% of compensation cases and 27.9% of criminal cases.^17^ In the study, fewer than half of the cases resulted in a first-instance finding of faulty medical practice against the defendant. The consistency between the findings and the acceptance rates reported in the literature suggests that primary care requires strengthening through mechanisms that enable continuous improvement—via effective and regular audits—of clinical practice protocols, documentation and oversight processes, training and reporting systems, workflow organization, and equipment such as visual guidance tools.

Expert opinions are frequently sought to assess whether a medical intervention was faulty and whether the harm sustained resulted from that intervention.^18^ In this framework, when reports submitted by courts and prosecutors are deemed insufficient or contradictory, a higher expert board may function as a reviewing body.^19^ In the study, an expert opinion was requested after the initial first-instance decision in 59.1% of cases. This relatively high proportion is important for ensuring objectivity in adjudication based on expert reports and for enhancing the reliability of decisions related to alleged malpractice.

National studies have shown high reversal rates by the Court of Cassation, indicating that malpractice cases often require reassessment during the judicial process.^7^^,^^20^^,^^21^ Similarly, in the study, 68.2% of first-instance decisions were reversed by the Court of Cassation. This finding is consistent with prior research and suggests that legal processes involving primary care practices frequently require a second review.

In the study, a statistically significant association was found between the type of malpractice allegation and first-instance court decisions. For example, acts such as “deficient medical intervention” and “incorrect prescribing” were generally assessed as fault, whereas incidents such as “cold-chain implementation error” or “incorrect injection” were more often assessed as involving no fault. Because complications may occur even when all steps of an injection procedure are performed correctly, and because cold-chain processes involve multiple stages—such as receipt at the health center, placement, temperature monitoring, and transfer—and because Article 7 of the Family Medicine Practice Regulation (“immunization services”) assigns responsibility for maintaining cold-chain conditions and taking necessary measures to both family physicians and family health staff, these processes are subject to complex regulatory arrangements; therefore, a tendency not to characterize such events as fault may be expected.^21^^,^^22^ Accordingly, in multi-stage health care processes, it may be a foreseeable outcome that errors are not always interpreted as direct fault.

A statistically significant association between the defendant’s professional role and first-instance court decisions was found, whereas no significant association was observed with the Court of Cassation decisions. First-instance courts frequently evaluated general practitioners as having committed malpractice while assessing nurses as not having done so; in subsequent proceedings, no significant pattern of change in judgments among physicians was observed, whereas judgments concerning nurses and midwives often changed. This may be related to factors such as the distribution of duties and responsibilities within the care team and the manner in which events were documented. More granular analyses are needed to clarify how evidence types—such as expert opinions, clinical records, and witness statements—and contextual factors shape these outcomes.

In malpractice litigation, the quality and content of expert reports can be decisive in fault assessment. Failures such as not ordering necessary tests, not requesting consultation in a timely manner, neglecting patient follow-up, or not performing required imaging are often considered fault.^5^ At this point, the role of expert reports is not limited to medical evaluation; they are also expected to be objective, clear, and scientifically grounded in a manner that contributes to determining responsibility within the legal framework. However, the literature frequently notes substantial variability in the content and presentation of expert opinions, and that some reports are inadequate both scientifically and legally.^23^ In 1 study examining malpractice lawsuits in Türkiye, all appealed criminal judgments were reversed by the Court of Cassation, most commonly due to insufficient or contradictory expert reports.^24^ Another study reported that 59% of appealed criminal judgments in malpractice cases were reversed, again most frequently due to incomplete/insufficient and contradictory expert reports.^5^ In the study, the relationship between the presence of an expert report and both first-instance and Court of Cassation decisions was examined, and no statistically significant differences were found at either level. While this difference was initially considered to reflect that expert reports may not have been sufficiently detailed or aligned with international standards for expert reporting, it is also important to consider that other types of evidence and contextual factors during ongoing proceedings may influence judicial outcomes.

In Conclusion, an examination of Court of Cassation decisions concerning primary health care indicates that medical malpractice is assessed largely within the framework of compensation claims. Nurses were the most frequently prosecuted professional group, and most allegations stemmed from incorrect injection administration. Due to high patient volumes, procedural diversity, and a broad spectrum of conditions, family health centers and health centers are particularly prominent in malpractice cases. In particular, post-injection sciatic nerve injuries underscore the need for safe practice and continuing professional education in primary care.

While physician-related individual omissions—such as deficiencies in medical intervention and incorrect prescribing—were generally deemed fault by courts of first instance, errors arising in multi-stage, system-based processes involving multiple health care personnel—such as “cold-chain implementation errors” or “incorrect injection”—were not considered fault. Nevertheless, to reduce malpractice in primary health care, it is important to adopt a comprehensive approach that strengthens training, supervision, documentation, communication, and the sharing of responsibilities.

## Figures and Tables

**Table 1.** General Information Regarding Allegations of Medical Malpractice

Groups		n (%)
Type of lawsuit	Criminal	10 (45.5)
	Compensation (civil damages)	12 (54.5)
Party filing the lawsuit	Physician	6 (27.3)
	Patient	5 (22.7)
	Patient’s relative	6 (27.3)
	Healthcare institution	5 (22.7)
Person involved	Family physician	7 (31.8)
	General practitioner	4 (18.2)
	Nurse	9 (40.9)
	Midwife	2 (9.1)
Reason for the alleged medical malpractice	Lack of heel-prick blood screening	1 (4.5)
	Deficiency in medical intervention	3 (13.6)
	Delay in establishing a diagnosis	1 (4.5)
	Incorrect vaccination	1 (4.5)
	Incorrect injection	7 (31.8)
	Cold-chain management error	4 (18.2)
	Incorrect report writing	2 (9.1)
	Incorrect prescription writing	3 (13.6)
Outcome of the incident	No harm	7 (31.8)
	Disability	1 (4.5)
	Injury	7 (31.8)
	Death	3 (13.6)
	Financial loss	4 (18.2)
Primary care facility where the incident occurred	Health center/Family Health Center	19 (86.4)
	Tuberculosis dispensary	3 (13.6)
Decision of the court of first instance	Conviction	10 (45.5)
	Acquittal	12 (54.5)
Expert opinion	Present	13 (59.1)
	Absent	9 (40.9)
Court of Cassation (Supreme Court) decision	Judgment upheld	7 (31.8)
	Judgment reversed	15 (68.2)
Total		22 (100)

**Table 2.** Relationship Between Alleged Grounds for Medical Malpractice and Decisions of the Court of First Instance and the Court of Cassation

Alleged Grounds for Medical Malpractice	Decision of the Court of First Instance			Decision of the Court of Cassation		
	Fault Present (n, %)	No Fault (n, %)	*P*	Judgment Upheld (n, %)	Judgment Reversed/Amended (n, %)	*P*
Inadequate heel-prick (newborn) screening	1 (4.5)	0	.021	0	1 (4.5)	.474
Inadequate medical intervention	3 (13.6)	0		0	3 (13.6)	
Delay in diagnosis	0	1 (4.5)		1 (4.5)	0	
Incorrect vaccination	1 (4.5)	0		0	1 (4.5)	
Incorrect injection	1 (4.5)	6 (27.3)		2 (9.1)	5 (22.7)	
Cold-chain management error	0	4 (18.2)		1 (4.5)	3 (13.6)	
Erroneous report writing	1 (4.5)	1 (4.5)		1 (4.5)	1 (4.5)	
Incorrect prescription writing	3 (13.6)	0				

**Table 3.** Relationship Between the Defended/Accused Party and the Decisions of the Court of First Instance and the Court of Cassation

Person Involved	Decision of the Court of First Instance			Decision of the Court of Cassation		
	Fault Present n (%)	No Fault n (%)	*P*	Judgment Upheld (n, %)	Judgment Reversed/Amended (n, %)	*P*
Family physician	3 (13.6)	4 (18.2)	.009	4 (18.2)	3 (13.6)	.145
General practitioner	4 (18.2)	0		2 (9.1)	2 (9.1)	
Nurse	1 (4.5)	8 (36.4)		1 (4.5)	8 (36.4)	
Midwife	2 (9.1)	0		0	2 (9.1)	

**Table 4.** Comparison of Expert Witness Opinions with Decisions of the Court of First Instance and the Court of Cassation

**Expert Opinion**	Decision of the Court of First Instance			Decision of the Court of Cassation		
	**Fault Present (n, %)**	**No Fault (n, %)**	***P***	**Judgment Upheld (n, %)**	**Judgment Reversed/Amended (n, %)**	***P***
Present	4 (18.2)	9 (40.9)	.096	4 (18.2)	9 (40.9)	.899
Absent	6 (27.3)	3 (13.6)		3 (13.6)	6 (27.3)	

## Data Availability

The data that support the findings of this study are available on request from the corresponding author.

## References

[1] T.C. sağlık bakanlığı halk sağlığı Genel müdürlüğü, aile Hekimliği uygulama ve geliştirme dairesi başkanlığı. Birinci basamak sağlık hizmeti; Published 2025. Accessed October 5, 2025. https://hsgm.saglik.gov.tr/tr/aile-hekimligi/bbsh.html.

[2] The Joint Commission on Accreditation of Healthcare Organizations (JCAHO). Sentinel events statistics. Jt Comm Perspect. 2006;26(3):8. Accessed October 1, 2025. www.jointcommission.org/Library/TM_Physicians/tmp_11_06.htm. 16933568

[3] Türk Dil Kurumu (TDK). Büyük Türkçe Sözlük; Tıp Terimleri Sözlüğü ; Published 2022. Accessed September 25, 2025. http://sozluk.gov.tr/.

[4] Şahbaz G Yücesoy H Akın Ö Malpraktis ve sağlık profesyonellerinin sorumlulukları. Sağlık Bilimleri Üniv Hemşirelik Derg. 2022;4(2):85 90. (doi: 10.48071/sbuhemsirelik.1012864)

[5] Yargıtay’da KM. Yılları Arasında Karara Bağlanan Hatalı Tıbbi Uygulama (Malpraktis) Dava Kararlarının Değerlendirilmesi [Uzmanlık Tezi]. Çukurova Üniversitesi Tıp Fakültesi Adli Tıp Anabilim Dalı. Adana, Türkiye; 2018:10.

[6] Kahyaoğlu K. Yılları arasında Yargıtay’a İntikal etmiş malpraktis Dosyalarının sağlık yönetimi açısından analizi. Ordu Üniversitesi Sağlık Bilimleri Enstitüsü, Sağlık Yönetimi Ana Bilim Dalı; 2022:8. Ordu, Türkiye.

[7] Aktürk G Özesen TA. 2021 yılında Yargıtay tarafından karara bağlanan hatalı tıbbi uygulama iddiası olgularının değerlendirilmesi. Adli Tıp Derg. 2023;37:19 25.

[8] Güzel S Yavuz MS Aşırdizer M. Adli Tıp Kurumu İhtisas Kurulları ile Yüksek Sağlık Şurası raporları arasında çelişki bulunan ve Adli Tıp Genel Kurulunda görüşülen malpraktis olgularının irdelenmesi. Bull Leg Med. 2002;7(1):14 20. (doi: 10.17986/blm.200271468)

[9] Özesen TA Kaya K. Dicle Tıp Derg. Çukurova Üniversitesi Tıp Fakültesi Adli Tıp Anabilim Dalı’na hatalı tıbbi uygulama iddiası ile başvuran olguların değerlendirilmesi (2015-2019). 2021;48:829 838.

[10] Kıvrak S Ölçeğinde K. Tıbbi Uygulama Hatası İddialarının Değerlendirilmesi [Uzmanlık Tezi]. Erciyes Üniversitesi Tıp Fakültesi Adli Tıp Anabilim Dalı. Kayseri, Türkiye; 2014:35.

[11] Elbüken B Profesyonellerine S. Yönelik Tıbbi Uygulama Hata İddiası İle Yüksek Sağlık Şurasına Gönderilen Olguların İrdelenmesi [Yüksek Lisans Tezi]. Marmara Üniversitesi. İstanbul, Türkiye; 2010:50.

[12] Çom U Üzün İ Gümüş B. Ölümle sonuçlanan kadın hastalıkları ve doğum olgularında tıbbi uygulama hatası iddialarının değerlendirilmesi. J Contemp Med. 2020;10(4):567 572. (doi: 10.16899/jcm.746800)

[13] Çırpı F Merih YD Kocabey MY. Hasta güvenliğine yönelik hemşirelik uygulamalarının ve hemşirelerin bu konudaki görüşlerinin belirlenmesi. Maltepe Üniv Hemşirelik Bilim Sanatı Derg. 2009;2(3):26 34.

[14] Doruk Analan P. Enjeksiyona sekonder siyatik nöropati hastalarımıza ait elektronöromiyografik sonuçlar: retrospektif bir değerlendirme. JPMR Sci. 2017;20:9 15.

[15] Kaya K Çekin N. Enjeksiyon sonrası gelişen nöropati: komplikasyon/malpraktis ayrımında ince bir çizgi. Kahramanmaraş Sütçü İmam Üniversitesi Tıp Fakültesi Dergisi. 2018;13(2):63 66. (doi: 10.17517/ksutfd.394213)

[16] Fapojuwo OA Akinlade TS Gbiri CA. A three year review of sciatic nerve injection palsy in the Physiotherapy Department of a Nigerian Specialist Hospital. Afr J Med Med Sci. 2008;37(4):389 393. 19301718

[17] Demir MF. Yılları Arasında Yargıtay’da Karara Bağlanan Tıbbi Uygulama Hatası Dava Dosyalarında Bilirkişi Raporlarının İçeriklerinin ve Kararlara Etkisinin Adli Tıbbi Açıdan Değerlendirilmesi [Tıpta Uzmanlık Tezi]. Selçuk Üniversitesi Tıp Fakültesi Adli Tıp Anabilim Dalı. Konya, Türkiye; 2024:50 100.

[18] Polat O Pakiş I. Tıbbi uygulama hatalarında hekim sorumluluğu. Acıbadem Üniv Sağlık Bilimleri Derg. 2011;2(3):119 125.

[19] Yazıcı YA Şen H Aliustaoğlu S Evaluation of the medical malpractice cases concluded in the General Assembly of Council of Forensic Medicine. Ulus Travma Acil Cerrahi Derg. 2015;21(3):204 208. (doi: 10.5505/tjtes.2015.24295) 26033654

[20] Kaya B Erkal E. Yargıtay’da karara bağlanan hemşire tıbbi uygulama hatası iddialarının adli tıbbi değerlendirilmesi: 20 yıllık retrospektif çalışma. Turk Klin Adli Tıp Adli Bilimler Derg. 2025;22(3):133 140.

[21] Vural T Erbaş M. Evaluating the opinions of the Supreme Court and the council of state on “informed consent (assent)” in compensation cases filed with medical malpractice claim. Bull Leg Med. 2024;29(1):20 28. (doi: 10.17986/blm.1665)

[22] Doğru Kızılkaya E . Aile hekimliğinde disiplin hükümleri ve ihtar puanları. Turk Adalet Akad Derg. 2016;28:471 495.

[23] Doğan MB. Adli bilimlerde adli tıp ve adli tıp dışı alanların Türkiye’deki yapılanması ile ilgili sorunlar: iki rapor ile değerlendirme. Bull Leg Med. 2022;27(1):66 77. (doi: 10.17986/blm.1531)

[24] Savaş H, Yansıyan Y. Tıbbi Müdahale Hataları. 1st ed. Seçkin Yayıncılık. Ankara, Türkiye; 2009:279 287.

